# Editorial: Hot topics in diabetes and steatotic liver disease

**DOI:** 10.3389/fcdhc.2026.1779266

**Published:** 2026-01-21

**Authors:** Roxana Adriana Stoica

**Affiliations:** Doctoral School, “Carol Davila” University of Medicine and Pharmacy, Bucharest, Romania

**Keywords:** central adiposity, FIB4 score, high fiber diet, MASLD, NAFLD, obesity, type 2 diabetes

Non-alcoholic fatty liver disease (NAFLD), currently reframed as metabolic dysfunction-associated steatotic liver disease (MASLD), is one of the most prevalent chronic liver conditions worldwide, together with type 2 diabetes mellitus (T2DM). Emerging evidence challenges conventional paradigms by identifying non-traditional risk factors and novel intervention strategies effective across diverse age groups and clinical phenotypes. In this Research Topic, which included eighteen articles, three publications deepen our understanding of NAFLD/MASLD pathogenesis and management by highlighting distinct, but interconnected, domains: anthropometric indices in adolescents, dietary modification in adults with diabetes and MASLD, and lipoprotein composite markers in non-obese adults with MASLD.

## Body composition beyond BMI in adolescents

Cui et al. looked into the association between the weight-adjusted waist index (WWI) and NAFLD in U.S. adolescents using NHANES data with liver ultrasound transient elastography as steatosis assessment. Their analysis demonstrates a positive correlation between higher WWI (meaning higher central adiposity relative to body weight) and NAFLD, independent of traditional predictors like BMI. Particularly, the association was non-linear, with steeper risk increases below the 10.65 threshold and more pronounced in older male adolescents. Thus, adolescent visceral adiposity may be a critical early driver of hepatic fat accumulation even in the absence of clear global obesity. Such findings necessitate a further reevaluation of screening paradigms in youth and argue for the use of newer anthropometric indices and body composition analysis that capture fat distribution (1), rather than relying solely on BMI.

## Translating risk factors into action: dietary fiber as an intervention in middle-aged adults with T2DM and MASLD

What appears as a risk factor in adolescence becomes a disease that requires intervention in adulthood. Identification of MASLD patients at risk for progression to fibrosis must be matched with effective interventions. Chen et al. conducted a randomized, controlled trial assessing the impact of a high-fiber cereal meal intervention on liver fibrosis progression and glycemic parameters (HbA1c, fasting plasma glucose) in adults with T2DM and MASLD. Over 12 weeks, patients receiving 12 g or 24 g/day of added dietary fiber exhibited a significant decrease in HbA1c, non-invasive fibrosis markers (FIB-4), and liver stiffness, particularly in the higher-dose group. These changes occurred without significant BMI reduction, suggesting benefits beyond simple weight loss.

The mechanisms probably include modulation of insulin sensitivity, decrease of glycemic excursions, and gut microbiota dynamics (2). Further studies are needed to determine long-term fibrosis outcomes and clarify optimal fiber intake (quantity and quality). This trial provides encouraging evidence that this type of dietary modification can influence both metabolic and hepatic endpoints, even in chronic, established disease.

## New evidence in lean adults: composite lipid indices matter

Although most research on this topic has included obese populations, Wu et al. give a deeper understanding of a seemingly contrasting population - a cohort of non-obese Chinese adults with normal conventional lipid profiles. They observed that the lipoprotein combine index (LCI) is significantly associated with incident NAFLD over five years. The identification of a non-linear relationship between LCI and NAFLD risk accentuate that metabolic risk extends beyond traditional dyslipidemia and can manifest even in low-risk individuals (normal weight). The LCI has the advantage of integrating multiple lipoprotein measures, capturing lipid metabolism perturbations that individual lipid fraction analysis might miss.

The adolescent and lean adult population studies mentioned above highlight a central theme: MAFLD risk exists on a continuum and may not be fully captured by conventional clinical markers such as BMI or individual lipid fractions. Integrating combined indices that reflect body composition and/or lipoprotein patterns offers a more personalized approach to identifying at-risk individuals across ages and phenotypes.

## Conclusion

For clinicians, these findings call for (1) expanded screening criteria for MASLD in lean adults, youth, and even children, which incorporate advanced anthropometric or composite biomarkers in risk assessment, and (2) early dietary counseling emphasizing fiber intake for individuals at risk or with established MASLD. For researchers, the next steps include (1) validating these indices in other populations, (2) integrating them into predictive algorithms, and (3) conducting long-term trials on dietary and lifestyle interventions in MASLD. The overall goal is to prevent progression and reduce the burden of liver-related morbidity and mortality through personalized, evidence-based care.
